# Comparison of the Efficacy and Safety of Temporary Spinal Cord Stimulation versus Pulsed Radiofrequency for Postherpetic Neuralgia: A Prospective Randomized Controlled Trial

**DOI:** 10.1155/2022/3880424

**Published:** 2022-10-11

**Authors:** Xiaohong Li, Pan Chen, Jian He, Xiang Huang, Dacheng Tang, Lumiao Chen, Xiaoping Wang

**Affiliations:** ^1^Department of Pain Management, The First Affiliated Hospital, Jinan University, Guangzhou 510630, Guangdong, China; ^2^Department of Pain Management, The First People's Hospital of Foshan, Foshan 528000, Guangdong, China

## Abstract

**Objectives:**

The objective of this study is to compare the safety and effectiveness of the temporary spinal cord stimulation (SCS) versus pulsed radiofrequency (PRF) in treating postherpetic neuralgia (PHN).

**Methods:**

From September 1, 2019, to May 30, 2020, 44 PHN patients admitted to the Pain Department of the Foshan First People's Hospital, China were enrolled in this study. The patients were randomly assigned to SCS and PRF groups in a ratio of 1 : 1 and were given respective therapy for 8 days. Rash, in all patients, was located in the trunk and extremities of the spinal nerve (C4-L5), and the pain intensity was greater than or equal to 7 points on the VAS scale. Subsequently, we evaluated the visual analogue scale (VAS), efficiency rate (ER), complete remission rate (CRR), daily sleep interference score (SIS), patient health questionnaire (PHQ-9), generalized anxiety disorder assessment (GAD-7), bodily pain (BP), and physical function (PF) sections of the 36-item short-form health survey (SF-36) at the following time points: presurgery, as well as 1 week, 1 month, 3 months, and 6 months postsurgery.

**Results:**

The final analysis was performed on 40 patients (*n* = 20 SCS cohort, and *n* = 20 PRF cohort). Both cohorts exhibited comparable baseline values (*P* > 0 : 05). Particularly, they were similar in age, sex, pain duration, involved dermatome, and comorbidity. Among the variables that demonstrated marked improvements from presurgical data to 1 week postsurgery were VAS, ER, CRR, SIS, PHQ-9, GAD-7, as well as BP and PF of the SF-36 in both cohorts. In addition, this improvement persisted for 6 months. There was no complication related to surgery in any of our patients.

**Conclusion:**

Based on our analysis, SCS exhibited better efficacy and safety than PRF. This study was prospectively registered in the Chinese Clinical Trial Registry (ChiCTR2100050647).

## 1. Introduction

Postherpetic neuralgia (PHN) is the leading and intractable complication of herpes zoster (HZ) [1–3]. The pain is characterized by a constant burning or stabbing sensation, and some individuals experience allodynia, and the symptoms may last for months or even years. Most patients also suffer from depression and anxiety, which negatively impact their quality of life [4]. According to statistics, 12.5% of HZ patients, aged ≥50 years, suffer from PHN for an average of 3 months, and 4-13% of patients experience PHN for 6 months. In addition, the proportion of affected individuals increases greatly with age [5, 6].

The major treatment options for PHN therapy according to the European Federation of Neurological Societies are pharmacological and invasive intervention-based approaches [7–9]. The invasive treatments include subcutaneous injection of the botulinum toxin A or triamcinolone, peripheral nerve stimulation, transcutaneous electrical nerve stimulation, stellate ganglion block, paravertebral block, pulsed radiofrequency, and spinal cord stimulation [10]. As most interventional procedures are nonspecific, the evidence employed for the treatment of PHN is Level 2, according to “The Oxford Levels of Evidence 2”. Hence, these approaches only receive grade B endorsement [10].

Radiofrequency is a minimally invasive, target-specific procedure that effectively reduces various types of persistent pain and has been used for PHN-related pain relief [11–13]. However, its application must be assured at the acute phase of PHN, as it shows poor therapeutic effects in patients with chronic PHN [14]. Spinal cord stimulation (SCS) is another well-established method of pain control and is provided by placing leads connected to pulse generators into the epidural space. This procedure is successfully applied for managing chronic neuropathic pain [15], failed back surgery syndrome (FBSS), and complex regional pain syndrome (CRPS) [16–18]. Moreover, it produces excellent results in reducing pain, improving function, and enhancing the patient's quality of life [19]. However, only a limited number of prospective randomized controlled trials (RCTs) assessed the impact of SCS in managing PHN. This study was designed to compare the efficiency and safety of SCS with PRF in treating PHN.

## 2. Materials and Methods

### 2.1. Participant Recruitment and Study Design

We conducted this prospective RCT from September 1, 2019, till May 30, 2020, to evaluate the safety and efficiency of SCS versus PRF in treating PHN. The study was approved by the ethical committee of the Foshan First People's Hospital, China and was registered with the Chinese Clinical Trial Registry (registration number: ChiCTR2100050647). All the procedures during this study were followed according to the guidelines of the Declaration of Helsinki. A total of 50 patients were screened based on our inclusion and exclusion criteria, and 44 patients were enrolled in the study. Patients' inclusion and exclusion were based on the criteria mentioned in Table 1. The discontinuation and elimination criteria of the subjects are shown in Table 2.

**Figure 1:**
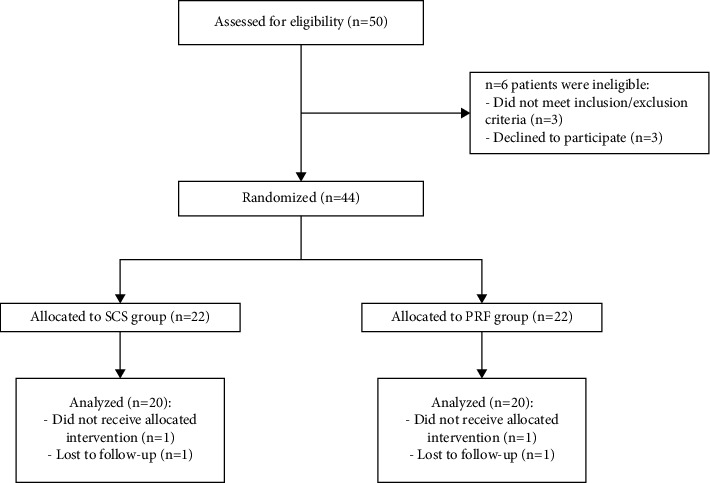
A flowchart of recruitment, randomization, and analyses.

In total, 50 patients were screened patients, and 6 patients were not allowed to participate in this study either due to inclusion/exclusive criteria (3 patients) or lack of providing consent to participate in this research (3 patients). A blinded statistician generated a random sequence, using an arbitrary number generator, and placed the numbers in numbered sealed and opaque envelopes. A nurse then used these envelopes to separate the remaining 44 patients into two cohorts, namely, SCS and PRF cohorts, in a 1 : 1 ratio. Before enrollment, all patients provided written informed consent. At the 6 month follow-up, 4 patients did not undergo the assigned treatments because 1 patient developed pneumothorax complication, 1 patient removed the electrode, and 2 patients did not participate in the follow-up. Thus, the study's final analysis only included 40 patients (SCS, *n* = 20 and PRF, *n* = 20) as shown in Figure 1.

### 2.2. Study Protocol

The included PHN confirmed patients were questioned by a nurse on the first day of treatment using a detailed questionnaire and were started a conventional drug regimen containing Pregabalin 75 mg, two times a day, and mecobalamin 0.5 mg, three times a day. In case of the emergence of adverse drug-related side effects, such as vomiting, nausea, dizziness, gastrointestinal discomfort, urinary retention, and constipation, symptomatic treatment was provided, and the adverse event was recorded.

Stimulus electrode and electric pulse generator (1^*∗*^8 compact 3778-75; Medtronic, Minneapolis, MN) was used in the SCS procedure while the Cosman G4 (Cosman Medical, Burlington, MA) and radiofrequency needle (10 cm; stericlin, Emmendingen, Germany) were used in the PRF procedure.

Patients undergoing SCS or PRF procedures were admitted for preoperative examination (Table 3). The patient's heart rate (HR), blood pressure (BP), and level of arterial oxygen saturation (SpO_2_) were monitored; and wide-awake local anesthesia was used.

**Figure 2:**
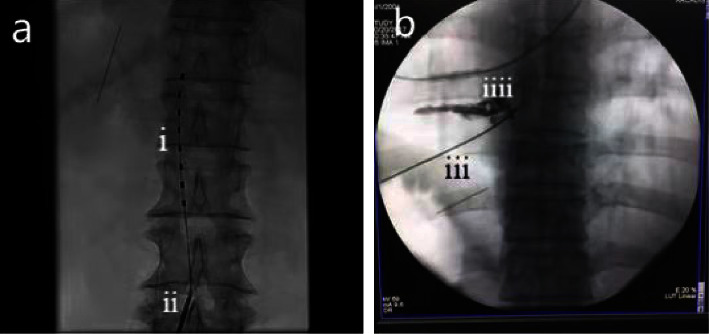
Representative X-ray images taken inside the digital subtraction angiography room; (a) a guided spinal cord stimulation electrode placement in the epidural cavity: (i) spinal cord stimulation electrode and (ii) epidural cavity puncture needle. (b) A guided radiofrequency needle placement in the target nerve, (iii) a radiofrequency needle, and (IV) thoracic spinal nerve under contrast.

The SCS procedure for all patients in the SCS group was carried out. Briefly, the patient was placed in a prone position. The puncture point, located in the intervertebral space, was disinfected and an aseptic scarf was laid out. Once the lidocaine local anesthesia took effect, a tunnel needle was held at an angle of about 30° to the skin, and then, it slowly penetrated the epidural cavity. Subsequently, the needle core was pulled out, and an 8-contact electrode was placed via digital subtraction angiography (DSA). The electrode position was then adjusted in the epidural cavity slightly to the left or right as needed, prior to the activation of the test mode. The parameters for voltage, frequency, and pulse width were adjusted as 2V, 40 Hz, and 210 us, respectively. Finally, the skin incision was sutured and fixed with a sterile gauze dressing, followed by hospitalization for 7 days after SCS treatment. The SCS procedure was completed in the DSA room, and the relevant images are presented in Figure 2(a).

For the PRF procedure, the puncture point was located at the inferior border of the cervical/thoracic/lumbar pedicle left/right anterior oblique position of DAS, and it was the lower margin of the upper vertebral body and the upper articular process of the corresponding segment. The shortest radiofrequency needle was inserted, and upon reaching the target point, a curved radiofrequency needle was introduced, along with the cannula, to enter the cervical/thoracic/lumbar superior articular process. The radiofrequency needle was then rotated inward into the intervertebral foramen. The needle tip depth was determined via positive and lateral fluoroscopy, and it was connected to the radio frequency machine. Subsequently, a sensory 50 HZ test was employed, where a voltage of 0.2 MV caused the patient's innervation area stimulated. The sensation of the original pain area was replicated with an exercise 2 HZ test, and the pulsed radio frequency mode was turned on. The radiofrequency needle was removed at the end of the procedure, and the wound was bandaged under mild pressure after 42° for 120 seconds and two cycles. The relevant images are presented in Figure 2(b). Following the procedure, the patients were observed and treated for 7 additional days in the hospital.

### 2.3. Evaluation and Outcome

We analyzed patient profiles, such as gender, age, pain duration, dermatome involved, comorbidity, and baseline information of patients at the time of admission. Moreover, we also recorded additional presurgical baseline data, such as visual analogue scale (VAS), daily sleep interference score (SIS), patient health questionnaire (PHQ-9), generalized anxiety disorder evaluation (GAD-7), bodily pain (BP), physical function (PF) of the 36-item short-form health survey (SF-36), effective rate (over 50% pain relief was considered effective regimen, ER), and complete remissions rate (postsurgical VAS <3 was considered complete remission, CRR). The major outcome was postoperative VAS, and secondary outcomes were SIS, PHQ-9, GAD-7, PF of SF-36, BP of SF-36 which were tested again at 1 week, 1 month, 3 months, and 6 months postsurgery.

For VAS pain assessment, a 10 cm line represented from 0-10 was used wherein, the ends 0 denoted no pain, and 10 denoted severe pain. Patients were requested to pick a point on the line that best described their degree of pain [20]. The daily sleep interference score was assessed by asking the patient to record how pain interfered with sleep, once the patient woke in the morning and after bedtime. 0 = no interference; 10 = unable to fall asleep [21]. PHQ-9 self-measurement scale was used for assessing patients' depression level where 0-4 = no depression, 5-9 = mild depression, 10-14 = moderate depression, 15-19 = moderate-severe depression, and 20-27 = severe depression criteria were adopted [22]. GAD-7 self-measurement scale was used to assess the anxiety level of patients where 0-4 = no anxiety disorder, 5-9 = mild anxiety disorder, 10-13 = moderate anxiety disorder, 14-18 = moderate-severe anxiety disorder, and 19-21 = severe anxiety disorder were followed [23]. The SF-36 with 8 domains having a scale of 0 (poor health) to 100 (good health) in each domain, for a total of 36 questions, was used to assess the health status of the patients. The validity, reliability, and applicability of the Chinese edition of the SF-36 were demonstrated previously [24]. Here, we monitored two areas, namely, PF and BP.

### 2.4. Sample Size Estimation

The main observation in this study was the degree of postoperative VAS pain reduction, and the overall sample size was estimated, based on the postoperative VAS. According to the pretest results of 14 samples, the mean postoperative VAS score was 2.0 in the SCS cohort and 3.2 in the PRF cohort, with a standard deviation of 1.23 points. In this study, a bilateral class I error *α* of 0.05 was set, and the test efficacy was 80%. The PRF cohort was set in a 1 : 1 ratio with the SCS cohort, and the sample population was predicted by the PASS 11.0 software (NCSS, United States), using a completely randomized design for the comparison of two samples' means. Based on the subsequent result, we required 18 samples per cohort, for a total of 36 cases. We considered an expected 20% loss to follow-up; therefore, 8 additional cases were proposed for this study; thus, providing a total sample size of 44 cases, with 22 in the PRF cohort and 22 in the SCS cohort.

### 2.5. Statistical Analysis

All data analyses were carried out through SPSS software version 26.0 and are presented as median (interquartile range) or percentage (%, count data). Those variables that did not conform to the normal distribution employed the Wilcoxon rank-sum test, and the count data was analyzed using the chi-square (*χ*^2^) or Fisher's exact test. *P* < 0 : 05 was set as the significance threshold.

## 3. Results

### 3.1. Patient Characteristics

Overall, 40 patients completed the 6 month follow-up, with 20 patients each in the SCS and PRP cohorts. We observed no obvious differences in the age, gender, pain duration, involved dermatome, and comorbidity between the two cohorts (*P* > 0 : 05), as shown in Table 4. In addition, the two cohorts also exhibited comparable preoperative VAS, SIS, GAD-7, BP, and PF of SF-36 (Table 4). The presurgical PHQ-9 in the SCS cohort was more severe than in the PRF cohort (*P*=0 : 012) (Table 4).

### 3.2. SCS Longitudinal Data

We recruited 20 patients in the SCS cohort, who participated in 6-month follow-up. We observed marked enhancements in the VAS, SIS, PHQ-9, GAD-7, BP, and PF of SF-36 1 week postsurgery, relative to the respective basal values (Table 5). The median (1st-3rd quartiles) VAS reduced significantly from 8.0 (8.0-8.3) to 2.0 (2.0-2.0), 1-week postsurgery (*P* < 0.001). Likewise, PHQ-9 reduced from 7.5 (6.0-9.3) to 3.5 (2.8-6.0) (*P* < 0.001), and GAD-7 reduced from 6.5 (4.5-9.5) to 3.0 (0.8-5.0) (*P*=0.008). Alternately, SIS enhanced markedly from 7.0 (7.0-8.0) to 0.0 (0.0-0.3) (*P* < 0 : 001). Similarly, the PF and BP portions of the SF-36 also exhibited marked increases from baseline 55.0 (40.0-70.0) and 31.0 (12.0–43.8) to 1-week postsurgery 87.5 (75.0-95.0, *P* < 0.001) and 52.0 (52.0-52.0, *P* < 0.001), respectively. In the SCS cohort, we observed considerable differences in VAS, SIS, PHQ-9, GAD-7, BP, and PF of SF-36 between baseline and postsurgical 1-, 3-, and 6-month values (Table 5).

### 3.3. PRF Longitudinal Data

The PRF cohort was composed of 20 hospitalized patients, who completed 6 months of follow-up. We observed marked enhancements in VAS, SIS, and PHQ-9 between baseline and 1-week postsurgery (Table 6). Similarly, there were marked improvements in PF of SF-36, from baseline 40.0 (40.0-56.3) to 80.0 (78.8-80.0) after 1-week postsurgery (*P* < 0.001) and from 40.0 (40.0-56.3) to 80.0 (78.8-80.0) after 1-month postsurgery (*P* < 0.001) (Table 6). In contrast, the VAS reduced from preoperative 8.0 (7.8-9.0) to postoperative 4.0 (4.0-5.0) (*P* < 0.001); SIS reduced from preoperative 7.0 (7.0-8.0) to postoperative 4.0 (4.0-4.3) (*P* < 0.001); and PHQ-9 reduced from preoperative 6.0 (5.0-6.0) to postoperative 4.0 (3.8-5.0) (*P*=0.04). Lastly, we observed no obvious difference in the GAD-7 and BP of SF-36 from baseline to 1 week, 1 month, 3 months, and 6 months after surgery. Moreover, there was also no marked difference in PF of SF-36 between baseline and at the 3- and 6-month follow-ups (Table 6).

### 3.4. Intergroup Comparison

**Figure 3:**
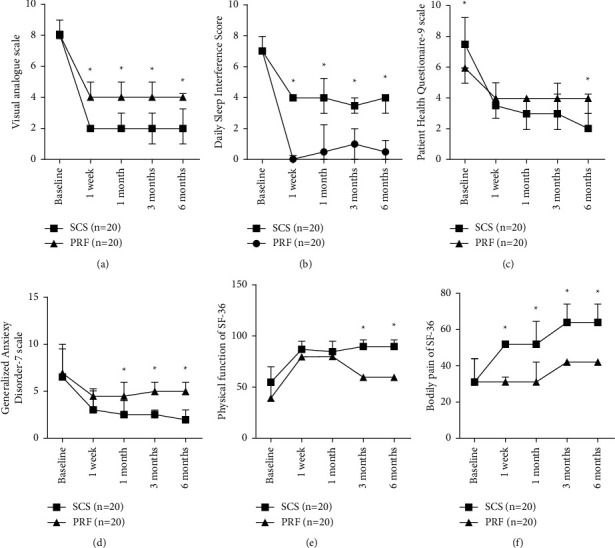
Comparison of the patient-reported results over time between the SCS (*n* = 20) and PRF (*n* = 20) cohorts (median, quartile). (a) Marked difference in VAS between the two cohorts at the 1 week, as well as 1-, 3-, and 6-months follow-ups. ^*∗*^*P* < 0.001. (b) Considerable difference in SIS between the two cohorts at the 1 week, as well as the 1-, 3-, and 6-month follow-ups. ^*∗*^*P* < 0.001. (c) Massive difference in PHQ-9 between the two cohorts at the 6-month follow-up, *P*=0.016. (d) Remarkable difference in GAD-7 between the two cohorts at the 1-, 3-, and 6-month follow-ups, ^*∗*^*P* < 0.001. (e) Considerable difference in PF of SF-36 between the two cohorts at the 3- and 6-month follow-ups, ^*∗*^*P* < 0.001. (f) Marked difference in BP of SF-36 between the two cohorts at the 1 week, as well as, at 1-, 3-, and 6-month follow-ups, ^*∗*^*P* < 0.001. Error bars refer to the 1st and 3rd quartiles.

Based on our analysis, at the 6-month follow-up, the VAS and SIS of the SCS cohort reduced remarkably, compared to the PRF cohort at 1 week (*P* < 0.001), 1 month (*P* < 0.001), 3 months (*P* < 0.001), and 6 months (*P* < 0.001) postsurgery (Figures 3(a) and 3(b)). The PHQ-9 in the SCS cohort decreased remarkably, relative to the PRF cohort at 6 months postsurgery (*P*=0.016). However, no obvious difference was noted in PHQ-9 between the two cohorts at 1 week (*P* > 0.05), 1 month (*P* > 0.05), and 3 months (*P* > 0.05) postsurgery (Figure 3(c)). The GAD-7 in the SCS cohort was markedly diminished, compared to the PRF cohort at 1 month (*P* < 0.001), 3 months (*P* < 0.001), and 6 months postsurgery (*P* < 0.001). However, it was nonsignificant at 1 week postsurgery (*P* > 0.05) (Figure 3(d)). The PF domain of SF-36 in the SCS cohort showed considerable enhancement, compared to the PRF cohort at 3 months (*P* < 0.001) and 6 months (*P* < 0.001). But, it was not significant at 1 week (*P* > 0.05) and 1 month postsurgery (*P* > 0.05) (Figure 3(e)). The BP domain of SF-36 in the SCS cohort showed considerable enhancement, relative to the PRF cohort at 1 week (*P* < 0.001), 1 month (*P* < 0.001), 3 months (*P* < 0.001), and 6 months postsurgery (*P* < 0.001) (Figure 3(f)). During the 6-month follow-up in this study, the EF and CRF were markedly elevated in the SCS cohort, compared to the PRF cohort at 1 week (*P* < 0.001), 1 month (*P* < 0.001), 3 months (*P* < 0.001), and 6 months postsurgery (*P* < 0.0011) (Tables 7 and 8).

### 3.5. Safety

We did not observe any complications or adverse effects within the 6-month follow-up duration following SCS or PRF.

## 4. Discussion

In this study, both SCS and PRF significantly improved the VAS score, enhanced analgesic efficiency, achieved complete relief, improved sleep quality, reduced anxiety and depression in patients, and increased PF and BP in SF-36. However, SCS provided better overall efficacy, compared to PRF. Moreover, the results from the 6-month follow-up confirmed that these interventions had prolonged positive effects. Furthermore, there were no reports of complications or adverse effects during the length of this study.

The outcomes of our research are consistent with several clinical trials which documented the efficacy of SCS in managing PHN [25–27]. In a prospective RCT by Botao Liu et al., 63 HZ patients over 50 years old were treated with SCS or PRF. Based on their research, the postoperative pain was significantly reduced, along with the analgesic usage, compared to before surgery. Moreover, this effect was sustained for one year, with no reported complication [25]. Moriyama and Dong also reported in their observational studies that SCS is an effective approach for the control of early HZ-related pain symptoms, and it prevents the transition from the acute to the chronic phase [26, 27]. In addition, several prospective RCTs confirmed the role of SCS in chronic neuropathic pain disorders, with unique effects in reducing pain, improving function, and enhancing the patient's quality of life [19, 28]. SCS is now widely used in a variety of chronic neuropathic pain treatments, particularly FBSS, CRPS, and other intractable neuropathic pain disorders [16, 18]. PHN is a typical neuropathic pain, and our trial demonstrated that SCS and PRF have promising application values in treating PHN. However, Cohen et al. revealed that the 3-month efficiency of PRF for chronic postoperative chest pain (CPTP) was only 6.7%, and the therapeutic effect of PRF gradually diminished with the duration of therapy. Moreover, the long-term analgesic effect was not obvious [29, 30]. In the present study, we demonstrated that SCS achieves better analgesic efficacy than PRF, and the longer duration of spinal nerve stimulation via SCS, relative to PRF, may be crucial to the efficacy of SCS.

We assessed all PHN patients in terms of PHQ-9, GAD-7, and SIS, and our results revealed that most patients exhibited varying degrees of anxiety and depression, as well as sleep disturbances. There was a drastic reduction in the PHQ-9, GAD-7, and SIS scores in the SCS and PRF cohorts after surgery, compared to the preoperative values. Several studies confirmed that neuropathic pain induces anxiety and depression in patients [31]. Robb collected data from 26 FBSS patients, who were treated with SCS, and assessed the 1-year postoperative hospital anxiety and depression scale (HADS) values. They reported that all participants exhibited a drastic decrease in the level of pain, along with a significant improvement in anxiety and depression on the HADS [32]. The improved emotion and enhanced sleep quality may be attributed to the augmented VAS scores at admission. Following a short SCS treatment cycle, the VAS score reciprocally decreases significantly, which, in turn, interrupts the vicious cycle of anxiety and depression and sleep-following disorders related to chronic pain.

In the current study, we demonstrated that the PF and BP in SF-36 improved significantly after surgery, with efficacy lasting for 6 months. The improvement was far more obvious in the SCS cohort, compared to the PRF cohort. As such, SCS was shown to remarkably enhance the quality of life of patients with neuropathic pain. In an international multicenter RCT involving 58 patients with diabetic peripheral neuropathy, Duarte reported EuroQol Five Dimensions Questionnaire (EQ-5D), with similar improvement to SF-36, in patients treated with SCS at the 6-month follow-up [33]. Schmader KE revealed that PHN often produces a negative effect on general daily activities, mental activities, well-being, social relationships, and sleep, due to the presence of chronic pain, and that the quality of life is decreased more significantly as the pain level increases [34]. Hence, improvements in PF and BP may be due to enhanced pain control.

During the follow-up period of this study, no major complications or adverse effects were reported for both SCS and PRF. Other side effects were mainly wound pain, skin pruritus, and constipation. Hematoma and infection are generally considered to be major complications of SCS, but neither occurred in this study.

SCS has been in practice for many years as a clinically effective, minimally invasive interventional treatment for chronic intractable neuropathic pain, with few side effects [26]. Approximately 30,000 chronic pain patients worldwide choose SCS for pain management each year [35]. SCS, as a neuromodulation therapy, has more advantages in treating neuropathic pain. The Neuromodulation Appropriateness Consensus Committee currently recommends SCS primarily for the treatment of FBSS and CRPS [15]. More prospective RCTs are warranted to further define the safety and efficacy of SCS in the treatment of peripheral nerve pain, residual limb pain, PHN, and other neuropathic pain due to nerve injury.

However, there is a lack of standard modality option for SCS in treating PHN, even though there are multiple SCS analgesic modalities. In addition to this, the cost of SCS treatment remains controversial. However, in the long term, the cost-effectiveness of SCS for PHN may be cheaper, despite appearing to be more expensive in the short term, compared to the PRF combination drugs.

The goal of our research was to investigate the short-term efficacy of short-course SCS or PRF for PHN. Our assessment time was set at 6 months. Therefore, our limitation in this study was the lack of long-term assessment. Hence, additional investigation involving a long-term follow-up study is warranted in coming years.

## 5. Conclusion

Both SCS and PRF can effectively treat herpes zoster neuralgia (HZ); however, SCS exhibited better efficacy than PRF.

## Figures and Tables

**Table 1:** Inclusion and exclusion criteria of PHN patients.

Inclusion criteria	Exclusion criteria
(1) Patients who met the diagnostic criteria agreed by the guidelines of PHN diagnosis and treatment	(1) Poor general condition, unable to objectively describe symptoms, unable to actively cooperate, or has a severe infection and respiratory insufficiency.

(2) Patients aged between 20 and 90 years, not limited to men or women	(2) Patients who suffered from allergic diseases or allergic reaction or who have drug allergy history

(3) Apart from the head and face, all spinal nerves innervated the trunk and limbs (C4-L5)	(3) Patients who ad epilepsy and/or family history of epilepsy

(4) With pain intensity VAS ≥7 points, had poor therapeutic effect, and required surgery	(4) Patients with severe systemic infection or HIV infection
	(5) Contraindications of SCS or PRF surgery
	(6) Severe peptic ulcer, pancreatitis, intestinal obstruction, and asthma

.	(7) Suspected or confirmed history of drug abuse
	(8) The investigator considered that there was a reason why the candidate should not be selected
	(9) Patients who could not follow the doctor's advice in terms of medication and surgical treatment or accept the visitation plan
	(10) Patients who could not understand the content of the questionnaire, or could not complete the questionnaire with the assistance of a visitor

SCS: spinal cord stimulation; PRP: pulsed radiofrequency; 1^st^-3^rd^: 1^st^-3^rd^ quartiles; VAS: visual analogue scale; HIV: human immunodeficiency virus.

**Table 2:** Discontinuation and elimination criteria of PHN patients.

Discontinuation criteria	Elimination criteria
(1) Subjects having serious adverse reactions during the study and it is not appropriate to continue participating in the trial	(1) Subjects do not meet the inclusion criteria, but they are included in the trial by mistake

(2) Subjects who face serious complications or deteriorating conditions during the study and emergency measures are urgently needed	(2) Subjects do not follow the prescribed treatment or have incomplete data that affects the efficacy evaluation and safety evaluation

(3) Subjects ask to quit the trial halfway	(3) Subjects with poor compliance and withdraw from the study by themselves

(4) Subjects have poor compliance and cannot comply with the study protocol	(4) Subjects received adjunctive treatments other than the intervention of this trial

**Table 3:** Threshold of preoperative exams that patients need to allow for procedure.

Index	Reference range
Blood routine	
White blood cell count (109/L)	4.00-10.0
Erythrocyte count (1012/L)	3.80-5.10
Hemoglobin (g/L)	115.00-150.00
Platelet count (109/L)	125.00-350.00
Neutrophil count (109/L)	1.80-6.30
Lymphocyte count (109/L)	1.10-3.20
Monocytes (109/L)	0.100.60
Eosinophil cells (109/L)	0.02-0.52

Hypersensitive C-reactive protein (mg/L)	0.00-10.00
Erythrocyte sedimentation rate (mm/h)	1.00-20.00

Coagulation function	
Prothrombin time (S)	10.00-14.00
Activated partial thromboplastin time (S)	23.30-32.50
Thrombin time (S)	13.00-25.00
Fibrinogen (g/L)	2.00-4.00

Procalcitonin (ng/mL)	0.00-0.06

Immune function	
Combined detection of human immunodeficiency	0.00-1.00
Virus antigen and antibody (the result is positive if	
The ratio is ≥ 1.0)	

**Table 4:** Baseline patient demographics.

	SCS cohort (*n* = 20)	PRF cohort (*n* = 20)	*P -*value
Age (y, median (1^st^–3^rd^))	65.5 (61.8-75.0)	63.5 (55.5-73.3)	0.369
Gender (M/F)	11/9	10/10	0.752
Pain duration (d, median (1st–3rd))	55.0 (35.0-71.25)	47.5 (30.0-67.5)	0.640

Involved dermatome (N/%)			
Cervical (N/%)	1 (5.0)	6 (30.0)	0.075
Thoracic (N/%)	15 (75.0)	9 (45.0)	
Lumbosacral (N/%)	4 (20.0)	5 (25.0)	

Comorbidity (N/%)	8 (40.0)	10 (50.0)	0.525
VAS (median (1^st^–3^rd^))	8.0 (8.0-8.3)	8.0 (7.8-9.0)	0.610
SIS (median (1^st^–3^rd^))	7.0 (7.0-8.0)	7.0 (7.0-8.0)	0.955
PHQ-9 (median (1^st^–3^rd^))	7.5 (6.0-9.3)	6.0 (5.0-6.0)	0.012
GAD-7 (median (1^st^–3^rd^))	6.5 (4.5-9.5)	7.0 (3.0-10.0)	1.000
PF of SF-36 (median (1^st^–3^rd^))	55.0 (40.0-70.0)	40.0 (40.0-56.3)	0.119
BP of SF-36 (median (1^st^–3^rd^))	31.0 (12.0-43.8)	31.0 (12.0-31.0)	0.429

SCS: spinal cord stimulation; PRP: pulsed radiofrequency; 1^st^-3^rd^: 1^st^-3^rd^ quartiles; VAS: visual analogue scale; SIS: daily sleep interference score; PHQ-9: patient health questionnaire-9; GAD-7: generalized anxiety disorder 7 scale; SF-36: the 36-item short-form health survey; PF: physical function; BP: bodily pain.

**Table 5:** Longitudinal results of the pain levels and quality of life for the SCS cohort over time.

Outcome	Time	SCS cohort (*n* = 20)	*P -*value^#^
VAS	Baseline	8.0 (8.0-8.3)	Ref
	1 week	2.0 (2.0-2.0)	<0.001
	1 month	2.0 (2.0-3.0)	<0.001
	3 months	2.0 (1.0-3.0)	<0.001
	6 months	2.0 (1.0-2.3)	<0.001
	*P -*value over time↑	<0.001	

SIS	Baseline	7.0 (7.0-8.0)	Ref
	1 week	0.0 (0.0-0.3)	<0.001
	1 month	0.5 (0.0-2.3)	<0.001
	3 months	1.0 (0.0-2.0)	<0.001
	6 months	0.5 (0.0-1.3)	<0.001
	*P -*value over time↑	<0.001	

PHQ-9	Baseline	7.5 (6.0-9.3)	Ref
	1 week	3.5 (2.8-6.0)	<0.001
	1 month	3.0 (2.0-4.0)	<0.001
	3 months	3.0 (2.0-5.0)	<0.001
	6 months	2.0 (2.0-3.0)	<0.001
	*P*-value over time↑	<0.001	

GAD-7	Baseline	6.5 (4.5-9.5)	Ref
	1 week	3.0 (0.8-5.0)	0.008
	1 month	2.5 (0.8-4.0)	0.004
	3 months	2.5(1.0-3.0)	<0.001
	6 months	2.0 (1.0-3.0)	<0.001
	*P*-value over time↑	<0.001	

PF of SF-36	Baseline	55.0 (40.0-70.0)	Ref
	1 week	87.5 (75.0-95.0)	<0.001
	1 month	85.0 (75.0-95.0)	<0.001
	3 months	90.0 (75.0-96.3)	<0.001
	6 months	90.0 (75.0-96.3)	<0.001
	*P*-value over time↑	<0.001	

BP of SF-36	Baseline	31.0 (12.0-43.8)	Ref
	1 week	52.0 (52.0-52.0)	<0.001
	1 month	52.0 (52.0-62.5)	<0.001
	3 months	64.0 (63.5-74.0)	<0.001
	6 months	64.0 (63.5-74.0)	<0.001
	*P*-value over time↑	<0.001	

SCS: spinal cord stimulation; 1^st^-3^rd^: 1^st^-3^rd^ quartiles; VAS: visual analogue scale; SIS: daily sleep interference score; PHQ-9: patient health questionnaire-9; GAD-7: generalized anxiety disorder 7 scale; SF-36: the 36-item short-form health survey; PF: physical function; BP: bodily pain. ^#^*P-*value comparison via post hoc or Wilcoxon signed-rank test. ↑*P-*value represents overall significance over time as per the Friedman test.

**Table 6:** Longitudinal results of the level of pain and quality of life in the PRF cohort over time.

Outcome	Time	PRF cohort (*n* = 20)	*P -*value^#^
VAS	Baseline	8.0 (7.8-9.0)	Ref
	1 week	4.0 (4.0-5.0)	<0.001
	1 month	4.0 (4.0-5.0)	<0.001
	3 months	4.0 (3.8-5.0)	<0.001
	6 months	4.0 (4.0-4.3)	<0.001
	*P -*value over time↑	<0.001	

SIS	Baseline	7.0 (7.0-8.0)	Ref
	1 week	4.0 (4.0-4.3)	<0.001
	1 month	4.0 (3.0-4.3)	<0.001
	3 months	3.5 (3.0-4.0)	<0.001
	6 months	4.0 (3.0-4.0)	<0.001
	*P*-value over time↑	<0.001	

PHQ-9	Baseline	6.0 (5.0-6.0)	Ref
	1 week	4.0 (3.8-5.0)	0.004
	1 month	4.0 (3.0-4.0)	<0.001
	3 months	4.0 (3.0-4.3)	<0.001
	6 months	4.0 (3.0-4.3)	<0.001
	*P*-value over time↑	<0.001	

GAD-7	Baseline	6.5 (4.5-9.5)	Ref
	1 week	4.5 (3.0-5.3)	0.108
	1 month	4.5 (4.0-6.0)	0.176
	3 months	5.0 (4.0-6.0)	0.208
	6 months	5.0 (4.0-6.0)	0.172
	*P*-value over time↑	0.114	

PF of SF-36	Baseline	40.0 (40.0-56.3)	Ref
	1 week	80.0 (78.8-80.0)	<0.001
	1 month	80.0 (60.0-80.0)	<0.001
	3 months	60.0 (55.0-60.0)	0.020
	6 months	60.0 (55.0-60.0)	0.020
	*P*-value over time↑	<0.001	

BP of SF-36	Baseline	31.0 (12.0-31.0)	Ref
	1 week	31.0 (31.0-33.8)	0.332
	1 month	31.0 (31.0-42.0)	0.256
	3 months	42.0 (41.8-42.0)	0.004
	6 months	42.0 (41.8-42.0)	0.004
	*P*-value over time↑	<0.001	

PRP: pulsed radiofrequency; 1^st^-3^rd^: 1^st^-3^rd^ quartiles; VAS: visual analogue scale; SIS: daily sleep interference score; PHQ-9: patient health questionnaire-9; GAD-7: generalized anxiety disorder 7 scale; SF-36: the 36-item short-form health survey; PF: physical function; BP: bodily pain. ^#^*P-*value comparison via post hoc or Wilcoxon signed-rank test. ↑ *P-*value represents overall significance over time as per the Friedman test.

**Table 7:** Comparison of efficiency rates between the SCS (*n* = 20) and PRF cohorts.

ER (N/%)	SCS cohort	PRF cohort	*P*-value^#^
Postsurgery	95	60	<0 : 001
1 week	85	50	<0 : 001
1 month	90	50	<0 : 001
3 months	100	65	<0 : 001
6 months	100	65	<0 : 001

SCS: spinal cord stimulation; PRP: pulsed radiofrequency; ER: efficiency rate; ^#^*P*-value comparison via post hoc or Wilcoxon signed-rank test.

**Table 8:** Comparison of the complete remission rate between the SCS (*n* = 20) and PRF cohorts.

CRR (N/%)	SCS	PRF	*P -*value^#^
Postsurgery	65	10	<0 : 001
1 week	85	15	<0 : 001
1 month	90	25	<0 : 001
3 months	100	10	<0 : 001
6 months	100	10	<0 : 001

SCS: spinal cord stimulation; PRP: pulsed radiofrequency; ER: efficiency rate;^#^*P-*value comparison via post hoc or Wilcoxon signed-rank test.

## Data Availability

Data supporting the results of this study are available to the *Department of Pain* Management, The First People's Hospital of Foshan, which can be contacted at awangolixiaohong@163.com.
